# Supplementary figures and images for: Clinical Progression in Alpha‐Synuclein Positive LRRK2‐PD and Sporadic Parkinson's Disease: A Longitudinal Analysis
Source: Mov Disord Clin Pract. 2026 Apr 19:10.1002/mdc3.70640. Online ahead of print. doi: 10.1002/mdc3.70640 (PMC13339648; doi:10.1002/mdc3.70640)

#
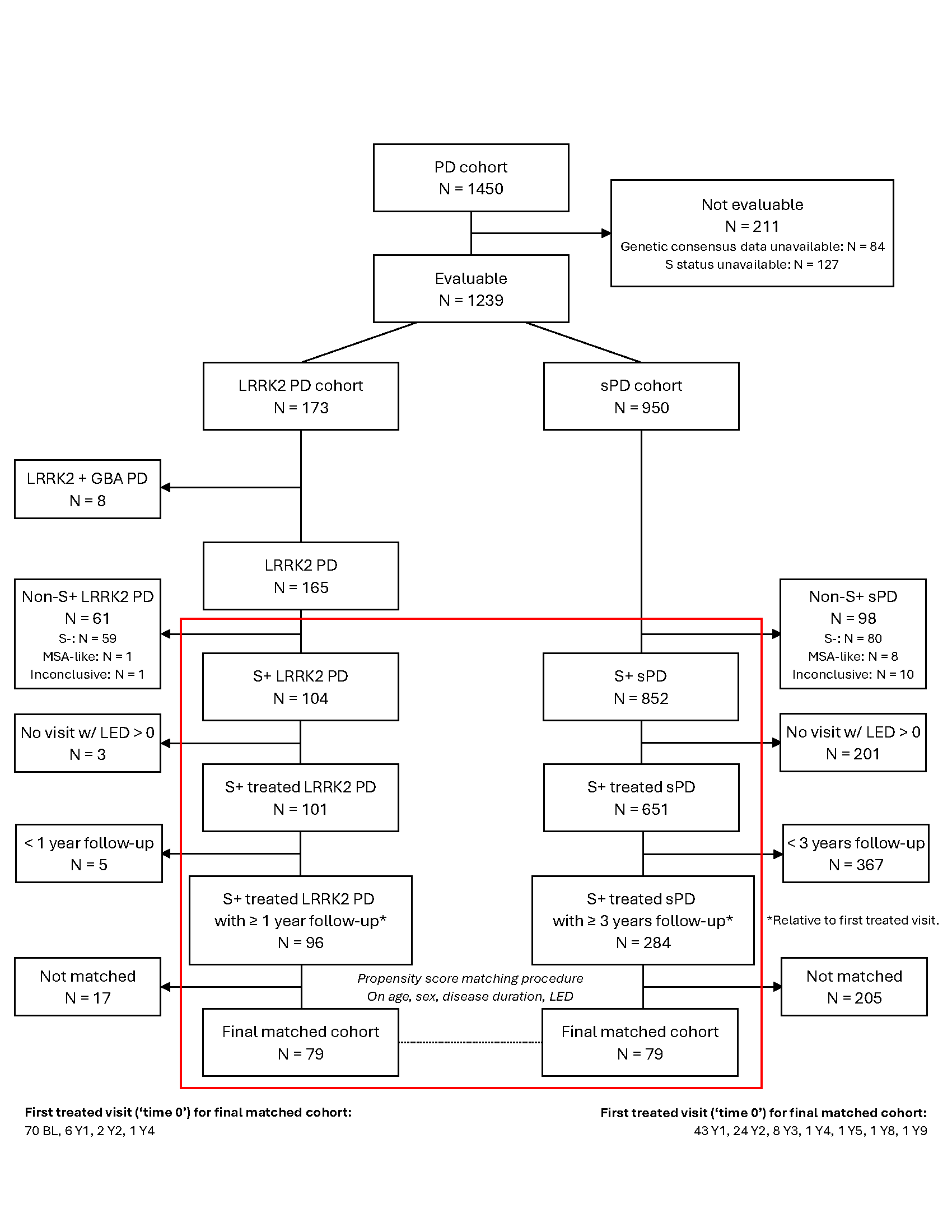

Supplement: Supplementary file 1 — Figure S1. Flowchart of participant selection for analysis population. LED, levodopa equivalent daily dose; MSA, multiple system atrophy; PD, Parkinson's disease; S−, alpha‐synuclein negative; S+, alpha‐synuclein positive; sPD, sporadic PD. [file MDC3-9999-0-s001.docx]
