# Supplementary material for: Clinical Progression in Alpha‐Synuclein Positive LRRK2‐PD and Sporadic Parkinson's Disease: A Longitudinal Analysis
Source: Mov Disord Clin Pract. 2026 Apr 19:10.1002/mdc3.70640. Online ahead of print. doi: 10.1002/mdc3.70640 (PMC13339648; doi:10.1002/mdc3.70640)
Supplement: Supplementary file 2 — TABLE S1. LED, levodopa equivalent dose (mg); PD, Parkinson's disease; S+; alpha‐synuclein positive. Baseline was defined to be a participant's first visit on treatment; LRRK2 and sporadic PD participants were required to have at least 1 and 3 years of follow‐up post‐treatment initiation, respectively, to be considered for matching. aComparisons by group used Chi‐Square or Fisher's Exact tests for categorical variables and Wilcoxon rank sum tests for continuous variables. TABLE S2. LED, levodopa equivalent dose; PD, Parkinson's disease; S+; alpha‐synuclein positive. aComparisons by group used Chi‐Square or Fisher's Exact tests for categorical variables and Wilcoxon rank sum tests for continuous variables. bFor the purposes of comparisons, APOE genotype was dichotomized as 0 vs. ≥ 1 e4 alleles. TABLE S3. Aβ1‐42, amyloid beta1‐42; MDS‐UPDRS, Movement Disorders Society—Modified Unified Parkinson's Disease Rating Scale; MoCA, Montreal Cognitive Assessment; NfL, neurofilament light chain; PD, Parkinson's disease; RBDSQ, REM Sleep Behavior Disorder Screening Questionnaire; S+; alpha‐synuclein positive; SCOPA‐AUT, Scales for Outcomes in Parkinson's disease–Autonomic Dysfunction. In cases where variables are missing less than 10% of values at all visits, the missing values are not shown. TABLE S4. MDS‐UPDRS, Movement Disorders Society—Modified Unified Parkinson's Disease Rating Scale; MoCA, Montreal Cognitive Assessment; PD, Parkinson's disease; RBDSQ, REM Sleep Behavior Disorder Screening Questionnaire; S+; alpha‐synuclein positive; SCOPA‐AUT, Scales for Outcomes in Parkinson's disease–Autonomic Dysfunction. Models were adjusted for levodopa equivalent dose at each visit. In cases where the model did not converge, the relevant fields are left blank. aFor binary outcomes, the time effect is reported as odds ratio (95% CI for OR). [file MDC3-9999-0-s003.docx]

**Supplementary Table 1. Sample demographics and other characteristics of S+ LRRK2-PD and S+ sporadic PD participants prior to matching.**

| **Variable** | **LRRK2-PD (N = 96)** | **Sporadic PD (N = 284)** | ***p*-value^a^** |
| --- | --- | --- | --- |
| **Age at baseline, years**, median (IQR) | 61.5 [54.9-66.8] | 63.7 [56.5-70.2] | 0.019 |
| **Male sex**, n (%) | 59 (61%) | 191 (67%) | 0.301 |
| **Years since PD diagnosis**, median (IQR) | 2.3 [1.2-4.2] | 1.7 [1.3-2.6] | 0.053 |
| **LED**, median (IQR) | 500.0 [300.0-705.0] | 246.2 [120.0-346.2] | <.001 |

# **Supplementary Table 2. Sample demographics and other characteristics of S+ LRRK2-PD participants at baseline and S+ sporadic PD participants at year 2 prior to matching.**

| **Variable** | **LRRK2 PD (N = 98)** | **Sporadic PD (N = 279)** | ***p*-value^a^** |
| --- | --- | --- | --- |
| **Age at baseline, years**, median (IQR) | 61.5 [54.7-66.9] | 64.2 [56.8-70.9] | 0.006 |
| **Age at PD symptom onset, years**, median (IQR) | 57.2 [47.8-62.1] | 60.1 [52.9-66.7] | 0.002 |
| Missing | 4 | 6 |  |
| **Male sex**, n (%) | 59 (60%) | 187 (67%) | 0.222 |
| **Years of education**, median (IQR) | 17.0 [14.0-19.0] | 16.0 [14.0-18.0] | 0.008 |
| **Years since PD diagnosis**, median (IQR) | 2.1 [1.1-4.0] | 2.4 [2.2-2.7] | 0.012 |
| **Race (% White)**, n (%) | 93 (95%) | 262 (94%) | 0.809 |
| Missing | 0 | 1 |  |
| **Hispanic**, n (%) | 14 (14%) | 7 (3%) | <.001 |
| **LED**, median (IQR) | 450.0 [250.0-700.0] | 300.0 [100.0-450.0] | <.001 |
| **LRRK2 variant**, n (%) |  |  |  |
| G2019S | 93 (95%) | - |  |
| R1441G | 4 (4%) | - |  |
| R1441C | 1 (1%) | - |  |
| **APOE genotype - number of e4 alleles^b^**, n (%) |  |  | 0.655 |
| 0 | 78 (80%) | 216 (77%) |  |
| 1 | 19 (19%) | 57 (20%) |  |
| 2 | 1 (1%) | 6 (2%) |  |

# **Supplementary Table 3. Longitudinal assessment of motor, non-motor, imaging and biofluid biomarkers of matched S+ LRRK2-PD and S+ sporadic PD participants.**

| **Variable** | **Baseline** | **Year 1** | **Year 2** | **Year 3** | **Year 4** |
| --- | --- | --- | --- | --- | --- |
|  | **LRRK2 N=79 sPD N=79** | **LRRK2 N=76 sPD N=76** | **LRRK2 N=62 sPD N=73** | **LRRK2 N=61 sPD N=74** | **LRRK2 N=59 sPD N=65** |
| **Modified Schwab and England**, mean (SD) | | | | | |
| LRRK2 PD | 92 (8) | 92 (8) | 91 (10) | 89 (10) | 89 (10) |
| sPD | 88 (7) | 86 (9) | 86 (9) | 85 (9) | 85 (10) |
| **Hoehn & Yahr stage (> 2) (ON)**, n (%) | | | | | |
| LRRK2 PD | 0 (0%) | 1 (1%) | 0 (0%) | 3 (5%) | 3 (5%) |
| Missing | 4 | 2 | 5 | 1 | 0 |
| sPD | 1 (1%) | 4 (6%) | 6 (8%) | 7 (10%) | 5 (8%) |
| Missing | 4 | 11 | 0 | 1 | 2 |
| **MDS-UPDRS I**, mean (SD) | | | | | |
| LRRK2 PD | 7 (5) | 8 (5) | 8 (4) | 9 (5) | 9 (5) |
| sPD | 7 (5) | 9 (6) | 9 (6) | 10 (7) | 10 (6) |
| **MDS-UPDRS II**, mean (SD) | | | | | |
| LRRK2 PD | 7 (4) | 8 (5) | 8 (5) | 9 (6) | 9 (6) |
| sPD | 8 (5) | 8 (6) | 9 (6) | 11 (7) | 11 (7) |
| **MDS-UPDRS III (ON)**, mean (SD) | | | | | |
| LRRK2 PD | 18 (10) | 17 (10) | 19 (10) | 19 (10) | 18 (9) |
| Missing | 5 | 2 | 7 | 3 | 6 |
| sPD | 23 (12) | 23 (14) | 24 (15) | 25 (14) | 26 (13) |
| Missing | 6 | 11 | 0 | 1 | 2 |
| **Gait (item 3.10) (ON) > 0**, n (%) | | | | | |
| LRRK2 PD | 45 (60%) | 47 (64%) | 35 (61%) | 35 (58%) | 35 (59%) |
| Missing | 4 | 2 | 5 | 1 | 0 |
| sPD | 51 (68%) | 43 (66%) | 49 (67%) | 53 (73%) | 47 (75%) |
| Missing | 4 | 11 | 0 | 1 | 2 |
| **Freezing of gait (item 3.11) (ON) > 0**, n (%) | | | | | |
| LRRK2 PD | 3 (4%) | 2 (3%) | 3 (5%) | 2 (3%) | 2 (3%) |
| Missing | 4 | 2 | 5 | 1 | 0 |
| sPD | 2 (3%) | 1 (2%) | 1 (1%) | 5 (7%) | 2 (3%) |
| Missing | 4 | 11 | 0 | 1 | 2 |
| **Tremor score (ON)**, mean (SD) | | | | | |
| LRRK2 PD | 3 (4) | 3 (3) | 3 (3) | 3 (3) | 2 (3) |
| Missing | 5 | 2 | 5 | 1 | 1 |
| sPD | 4 (4) | 3 (3) | 3 (3) | 4 (4) | 4 (4) |
| Missing | 4 | 11 | 0 | 1 | 2 |
| **Total MDS-UPDRS (ON)**, mean (SD) | | | | | |
| LRRK2 PD | 31 (15) | 33 (15) | 34 (15) | 36 (16) | 35 (14) |
| Missing | 7 | 3 | 7 | 3 | 6 |
| sPD | 38 (17) | 39 (21) | 42 (21) | 46 (21) | 47 (20) |
| Missing | 7 | 11 | 0 | 1 | 2 |
| **Geriatric Depression Scale**, mean (SD) | | | | | |
| LRRK2 PD | 3 (3) | 3 (3) | 3 (3) | 3 (3) | 3 (3) |
| sPD | 2 (3) | 3 (3) | 2 (2) | 3 (3) | 3 (3) |
| **State-Trait Anxiety Inventory**, mean (SD) | | | | | |
| LRRK2 PD | 67 (18) | 68 (20) | 67 (17) | 69 (21) | 67 (20) |
| sPD | 64 (17) | 66 (18) | 65 (18) | 65 (19) | 64 (18) |
| **SCOPA-AUT**, mean (SD) | | | | | |
| LRRK2 PD | 12 (7) | 13 (8) | 14 (8) | 14 (9) | 14 (8) |
| sPD | 11 (7) | 13 (8) | 13 (8) | 14 (8) | 14 (8) |
| **RBDSQ**, mean (SD) | | | | | |
| LRRK2 PD | 4 (2) | 4 (3) | 4 (3) | 4 (3) | 4 (3) |
| sPD | 4 (3) | 5 (3) | 5 (3) | 5 (3) | 5 (3) |
| **RBDSQ ≥ 6**, n (%) | | | | | |
| LRRK2 PD | 14 (18%) | 17 (22%) | 13 (22%) | 11 (18%) | 17 (29%) |
| sPD | 25 (32%) | 27 (36%) | 26 (36%) | 29 (39%) | 28 (43%) |
| **Epworth Sleepiness Scale**, mean (SD) | | | | | |
| LRRK2 PD | 7 (4) | 8 (4) | 8 (5) | 7 (5) | 8 (4) |
| sPD | 8 (4) | 7 (4) | 8 (5) | 8 (5) | 9 (5) |
| **MoCA**, mean (SD) | | | | | |
| LRRK2 PD | 27 (3) | 27 (3) | 27 (2) | 27 (2) | 27 (2) |
| Missing | 0 | 0 | 5 | 3 | 6 |
| sPD | 26 (3) | 26 (3) | 26 (3) | 26 (5) | 27 (3) |
| Missing | 2 | 2 | 1 | 0 | 1 |
| **Hopkins Verbal Learning Test Delayed Recall t-score**, mean (SD) | | | | | |
| LRRK2 PD | 46.3 (12.1) | 46.9 (11.9) | 48.0 (12.2) | 45.4 (13.0) | 46.0 (12.5) |
| Missing | 1 | 2 | 5 | 3 | 7 |
| sPD | 45.6 (11.8) | 44.3 (12.2) | 44.6 (13.5) | 45.1 (13.8) | 46.4 (13.0) |
| Missing | 3 | 1 | 1 | 1 | 3 |
| **Hopkins Verbal Learning Test Immediate/Total Recall t-score**, mean (SD) | | | | | |
| LRRK2 PD | 46.4 (11.1) | 47.7 (11.7) | 49.5 (10.3) | 45.7 (11.7) | 47.6 (11.0) |
| sPD | 43.7 (10.4) | 45.1 (12.9) | 46.4 (13.5) | 45.8 (12.9) | 47.1 (12.4) |
| **Benton Judgement of Line Orientation scaled score**, mean (SD) | | | | | |
| LRRK2 PD | 11.6 (3.2) | 11.1 (3.1) | 12.1 (2.3) | 10.9 (3.2) | 11.7 (3.0) |
| sPD | 12.2 (3.1) | 12.5 (2.9) | 11.9 (3.3) | 12.1 (3.1) | 12.0 (3.2) |
| **Letter Number Sequencing scaled score**, mean (SD) | | | | | |
| LRRK2 PD | 11.1 (2.9) | 11.0 (2.8) | 11.6 (2.8) | 11.2 (2.2) | 11.1 (2.6) |
| Missing | 2 | 2 | 5 | 4 | 7 |
| sPD | 11.0 (2.6) | 11.2 (2.8) | 10.8 (3.1) | 11.2 (3.3) | 11.3 (3.1) |
| Missing | 3 | 1 | 1 | 1 | 3 |
| **Symbol Digit Modalities Test t-score**, mean (SD) | | | | | |
| LRRK2 PD | 45.1 (9.8) | 46.6 (9.2) | 47.4 (9.4) | 48.4 (10.0) | 46.5 (8.5) |
| Missing | 1 | 1 | 5 | 3 | 6 |
| sPD | 44.7 (9.9) | 45.1 (11.8) | 45.1 (11.1) | 43.2 (11.2) | 44.9 (11.4) |
| Missing | 3 | 1 | 1 | 1 | 3 |
| **Semantic Fluency (Animals) t-score**, mean (SD) | | | | | |
| LRRK2 PD | 51.7 (12.1) | 53.9 (11.7) | 50.5 (10.8) | 52.1 (11.8) | 52.0 (12.8) |
| sPD | 50.3 (11.0) | 51.5 (10.9) | 50.2 (11.7) | 49.7 (11.7) | 51.6 (11.7) |
| **Cognitive Summary Score**, mean (SD) | | | | | |
| LRRK2 PD | -0.0 (0.7) | 0.0 (0.7) | 0.1 (0.6) | -0.0 (0.7) | 0.0 (0.6) |
| Missing | 3 | 2 | 5 | 4 | 8 |
| sPD | -0.1 (0.7) | -0.0 (0.8) | -0.1 (0.8) | -0.1 (0.8) | 0.0 (0.8) |
| Missing | 3 | 2 | 1 | 1 | 3 |
| **Number of Impulse Control Disorders ≥ 1**, n (%) | | | | | |
| LRRK2 PD | 28 (35%) | 33 (43%) | 20 (34%) | 22 (37%) | 17 (29%) |
| sPD | 18 (23%) | 19 (25%) | 15 (21%) | 23 (31%) | 18 (28%) |
| **Lowest putamen ratio**, mean (SD) | | | | | |
| LRRK2 PD | 0.30 (0.10) | 0.26 (0.04) | 0.25 (0.10) | 0.27 (0.02) | 0.22 (0.08) |
| Missing | 11 | 69 | 21 | 58 | 27 |
| sPD | 0.26 (0.08) | 0.25 (0.08) | 0.20 (0.09) | 0.22 (0.07) | 0.27 (N/A) |
| Missing | 19 | 30 | 57 | 38 | 64 |
| **Aβ_1-42_ (pg/mL)**, median [IQR] | | | | | |
| LRRK2 PD | 793.4 [639.9-1,030.7] | 875.0 [641.8-1,115.3] | 888.5 [616.7-1,121.4] | 795.0 [598.0-1,068.7] | 900.8 [560.3-1,237.3] |
| Missing | 8 | 24 | 25 | 33 | 40 |
| sPD | 814.8 [622.3-1,137.0] | 703.0 [540.5-1,107.0] | 768.0 [643.0-1,187.6] | 757.8 [586.9-1,260.9] | 608.5 [500.3-882.4] |
| Missing | 14 | 23 | 28 | 31 | 38 |
| **Aβ_1-42_ ≤ 683 pg/mL**, n (%) | | | | | |
| LRRK2 PD | 22 (31%) | 14 (27%) | 11 (30%) | 11 (39%) | 5 (26%) |
| Missing | 8 | 24 | 25 | 33 | 40 |
| sPD | 20 (31%) | 24 (45%) | 14 (31%) | 18 (42%) | 14 (52%) |
| Missing | 14 | 23 | 28 | 31 | 38 |
| **Aβ_1-42_ ≤ 710 pg/mL**, n (%) | | | | | |
| LRRK2 PD | 26 (37%) | 14 (27%) | 13 (35%) | 11 (39%) | 5 (26%) |
| Missing | 8 | 24 | 25 | 33 | 40 |
| sPD | 23 (35%) | 27 (51%) | 17 (38%) | 19 (44%) | 14 (52%) |
| Missing | 14 | 23 | 28 | 31 | 38 |
| **Total tau (pg/mL)**, median [IQR] | | | | | |
| LRRK2 PD | 140.1 [118.6-193.6] | 152.9 [119.7-186.4] | 140.1 [114.2-190.9] | 133.7 [107.9-176.3] | 138.9 [124.0-179.8] |
| Missing | 8 | 24 | 25 | 33 | 40 |
| sPD | 155.6 [122.7-210.2] | 147.3 [117.2-191.2] | 162.7 [132.6-208.0] | 156.5 [112.2-225.0] | 138.3 [106.9-162.0] |
| Missing | 14 | 22 | 27 | 31 | 38 |
| **Total tau ≥ 266 pg/mL**, n (%) | | | | | |
| LRRK2 PD | 4 (6%) | 6 (12%) | 3 (8%) | 1 (4%) | 1 (5%) |
| Missing | 8 | 24 | 25 | 33 | 40 |
| sPD | 4 (6%) | 3 (6%) | 4 (9%) | 4 (9%) | 2 (7%) |
| Missing | 14 | 22 | 27 | 31 | 38 |
| **Total tau ≥ 112 pg/mL**, n (%) | | | | | |
| LRRK2 PD | 60 (85%) | 46 (88%) | 28 (76%) | 20 (71%) | 18 (95%) |
| Missing | 8 | 24 | 25 | 33 | 40 |
| sPD | 53 (82%) | 42 (78%) | 38 (83%) | 34 (79%) | 20 (74%) |
| Missing | 14 | 22 | 27 | 31 | 38 |
| **Phospho-tau_181_ (pg/mL)**, median [IQR] | | | | | |
| LRRK2 PD | 12.3 [10.0-15.5] | 13.1 [10.0-16.3] | 13.9 [10.5-16.9] | 11.5 [9.4-14.5] | 13.9 [10.8-14.8] |
| Missing | 8 | 24 | 25 | 33 | 40 |
| sPD | 12.5 [9.8-17.3] | 12.0 [9.4-16.1] | 13.1 [10.2-17.5] | 12.2 [9.4-18.5] | 11.3 [9.3-13.6] |
| Missing | 14 | 22 | 27 | 31 | 38 |
| **Phospho-tau_181_ ≥ 24 pg/mL**, n (%) | | | | | |
| LRRK2 PD | 4 (6%) | 4 (8%) | 3 (8%) | 1 (4%) | 1 (5%) |
| Missing | 8 | 24 | 25 | 33 | 40 |
| sPD | 6 (9%) | 3 (6%) | 5 (11%) | 3 (7%) | 2 (7%) |
| Missing | 14 | 22 | 27 | 31 | 38 |
| **Phospho-tau_181_ ≥ 17.6 pg/mL**, n (%) | | | | | |
| LRRK2 PD | 12 (17%) | 12 (23%) | 8 (22%) | 5 (18%) | 3 (16%) |
| Missing | 8 | 24 | 25 | 33 | 40 |
| sPD | 15 (23%) | 13 (24%) | 10 (22%) | 11 (26%) | 5 (19%) |
| Missing | 14 | 22 | 27 | 31 | 38 |
| **Serum NfL (pg/mL)**, mean (SD) | | | | | |
| LRRK2 PD | 11.6 (4.2) | 14.3 (7.6) | 13.3 (5.1) | 13.3 (5.5) | 24.0 (3.1) |
| Missing | 20 | 20 | 22 | 30 | 56 |
| sPD | 13.6 (6.1) | 15.0 (7.8) | 15.1 (7.7) | 21.4 (23.6) | 18.6 (11.9) |
| Missing | 16 | 17 | 31 | 52 | 27 |

# **Supplementary Table 4. Results of linear mixed effects models in matched S+ LRRK2-PD and S+ sporadic PD participants.**

|  | **Linear Model^a^** | | | **Quadratic Model** | | **3-Way Interaction Model** | |
| --- | --- | --- | --- | --- | --- | --- | --- |
| **Variable** | **Interaction p-value** | **Time Effect (95% CI)** | **Time p-value** | **p-value** | **Effect** | **p-value** | **Effect** |
| Modified Schwab and England | 0.256 | -1.111 (-1.673, -0.550) | <0.001 | 0.591 | 0.264 | 0.219 | -1.399 |
| Hoehn & Yahr stage (> 2) (ON)^a^ |  |  |  |  |  |  |  |
| MDS-UPDRS I | 0.325 | 0.423 (0.140, 0.706) | 0.004 | 0.225 | 0.300 | 0.193 | 0.750 |
| MDS-UPDRS II | 0.391 | 0.890 (0.575, 1.205) | <0.001 | 0.702 | 0.105 | 0.509 | 0.414 |
| MDS-UPDRS III (ON) | 0.308 | 0.730 (-0.030, 1.489) | 0.060 | 0.125 | -0.897 | 0.675 | -0.655 |
| Gait (item 3.10) (ON) > 0^a^ | 0.105 | 1.001 (0.800, 1.252) | 0.993 | 0.955 | 0.013 | 0.275 | 0.508 |
| Freezing of gait (item 3.11) (ON) > 0^a^ | 0.436 | 1.221 (0.722, 2.064) | 0.455 | 0.448 | 0.454 | 0.592 | 0.840 |
| Tremor score (ON) | 0.049 | LRRK2: -0.231 (-0.521, 0.060) sPD: 0.161 (-0.118, 0.441) | LRRK2: 0.119 sPD: 0.257 | 0.122 | -0.279 | 0.430 | 0.337 |
| Total MDS-UPDRS (ON) | 0.069 | 2.040 (0.980, 3.099) | <0.001 | 0.483 | -0.574 | 0.556 | 1.283 |
| Geriatric Depression Scale | 0.399 | 0.007 (-0.147, 0.161) | 0.928 | 0.551 | -0.081 | 0.364 | -0.281 |
| State-Trait Anxiety Inventory | 0.818 | -0.404 (-1.450, 0.643) | 0.447 | 0.965 | 0.039 | 0.307 | -2.179 |
| SCOPA-AUT | 0.987 | 0.500 (0.085, 0.914) | 0.018 | 0.425 | -0.260 | 0.821 | -0.191 |
| RBDSQ | 0.227 | 0.044 (-0.106, 0.193) | 0.564 | 0.517 | 0.088 | 0.700 | -0.115 |
| RBDSQ ≥ 6^a^ | 0.863 | 1.108 (0.874, 1.406) | 0.396 | 0.330 | 0.250 | 0.264 | -0.755 |
| Epworth Sleepiness Scale | 0.416 | 0.332 (0.103, 0.561) | 0.005 | 0.115 | 0.358 | 0.222 | 0.559 |
| MoCA | 0.605 | 0.114 (-0.045, 0.272) | 0.158 | 0.523 | -0.089 | 0.644 | 0.149 |
| Hopkins Verbal Learning Test Delayed Recall t-score | 0.120 | -0.033 (-0.829, 0.763) | 0.935 | 0.821 | -0.169 | 0.293 | 1.706 |
| Hopkins Verbal Learning Test Immediate/Total Recall t-score | 0.414 | 0.181 (-0.551, 0.912) | 0.626 | 0.494 | 0.475 | 0.485 | 1.025 |
| Benton Judgement of Line Orientation scaled score | 0.083 | -0.058 (-0.240, 0.123) | 0.526 | 0.352 | -0.178 | 0.886 | -0.052 |
| Letter Number Sequencing scaled score | 0.555 | 0.003 (-0.152, 0.158) | 0.967 | 0.108 | -0.243 | 0.556 | -0.182 |
| Symbol Digit Modalities Test t-score | 0.358 | -0.117 (-0.629, 0.395) | 0.653 | 0.104 | -0.779 | 0.793 | -0.270 |
| Semantic Fluency (Animals) t-score | 0.806 | -0.223 (-0.854, 0.407) | 0.484 | 0.787 | 0.166 | 0.662 | 0.542 |
| Cognitive Summary Score | 0.902 | -0.007 (-0.040, 0.027) | 0.698 | 0.467 | -0.022 | 0.830 | 0.014 |
| Number of Impulse Control Disorders ≥ 1^a^ | 0.095 | 0.947 (0.771, 1.163) | 0.602 | 0.974 | 0.007 | 0.495 | -0.301 |
